# Foot progression angle asymmetry as a potential biomechanical marker of radiographic severity in knee osteoarthritis

**DOI:** 10.3389/fbioe.2025.1667271

**Published:** 2025-09-12

**Authors:** Jing Dai, Jian-Xiong Ma, Bin Lu, Hao-Hao Bai, Hongjie Zhang, Xin-Long Ma

**Affiliations:** ^1^ Biomechanics Labs of Orthopaedics Institute, Tianjin Hospital, Tianjin University, Tianjin, China; ^2^ Tianjin Key Laboratory of Orthopaedic Biomechanics and Medical Engineering, Tianjin, China; ^3^ The People’s Hospital Of Dehong Prefecture, Yunnan, China

**Keywords:** knee osteoarthritis, gait asymmetry, foot progression angle, compensatory mechanism, radiographic severity

## Abstract

**Introduction:**

Knee osteoarthritis (OA) induces asymmetric gait adaptations, yet the role of foot progression angle (FPA) remains unquantified. This study aimed to: (1) compare FPA differences between affected and contralateral limbs in knee OA patients, (2) characterize FPA asymmetry patterns, and (3) identify factors associated with inter-limb FPA differences.

**Methods:**

FPA asymmetry was quantified in 127 patients scheduled for unilateral high tibial osteotomy (HTO). FPA was measured bilaterally during natural walking. Multivariable logistic regression identified factors associated with asymmetry patterns.

**Results:**

FPA was significantly larger on contralateral limbs versus affected limbs (15.21° ± 7.72° vs 11.38° ± 8.13°, *p* < 0.001). Adjusted for covariates, patients with Kellgren-Lawrence (K&L) grade 1/2 OA had 70.2% lower odds (OR = 0.298, 95%CI:0.091–0.982) and grade 3 OA had 76.2% lower odds (OR = 0.238, 95%CI:0.081–0.700) of exhibiting contralateral-dominant FPA asymmetry compared to grade 4 OA (*P* < 0.05).

**Conclusion:**

Knee OA patients exhibit FPA asymmetry characterized by greater toe-out on the contralateral limb, correlating positively with higher radiographic severity (K&L grade) in the affected knee. FPA asymmetry may serve as a potential biomechanical marker of OA severity.

## Introduction

Osteoarthritis (OA), the most prevalent degenerative joint disease, is characterized primarily by degenerative changes in articular cartilage. Knee OA manifests clinically as pain, stiffness, and reduced range of motion (Sharma, 2021). According to recent Global Burden of Disease data, osteoarthritis ranks as the 12th leading cause of global disability burden worldwide, with knee OA contributing >80% of this burden (Collaborators, 2017). Pathogenesis involves a complex multifactorial process involving age, sex, body mass, and biomechanical determinants (Barbour et al., 2014). Biomechanical dysregulation has received growing research focus (Barnett, 2018; Hsu and Siwiec, 2022).

Given the critical role of aberrant knee biomechanics in OA pathogenesis, foot progression angle (FPA) modification (particularly increasing toe-out angle) has emerged as a promising conservative biomechanical intervention (Chang et al., 2007; Simic et al., 2013). FPA refers to the angle between the long axis of the foot (the line from the midpoint of the heel to the head of the second metatarsal) and the line of progression (the straight trajectory of the subject’s walking), with toe-out being positive. Knee adduction moment (KAM), a key biomechanical surrogate of medial compartment loading, is quantified as the vertical ground reaction force (vGRF) multiplied by its knee moment arm. This parameter strongly correlates with disease progression in knee OA (Komistek et al., 1999; Pollo et al., 2002). Implementing a toe-out gait reduces elevated KAM in OA patients. This reduction occurs through decreased frontal plane moment arm, resulting from lateral displacement of the center of pressure during stance phase (Reeves and Bowling, 2011).

Patients with knee OA frequently demonstrate inter-limb gait asymmetry due to disease pathophysiology (Creaby et al., 2012; Robadey et al., 2018). However, characterization of FPA asymmetry in knee OA remains limited. This study therefore aimed to: (1) quantify FPA differences between affected and contralateral limbs in knee OA patients, (2) characterize asymmetry patterns, and (3) identify factors associated with inter-limb FPA differences. We hypothesized significant FPA asymmetry would exist across affected and contralateral sides.

## Materials and methods

### Participants

**FIGURE 1:**
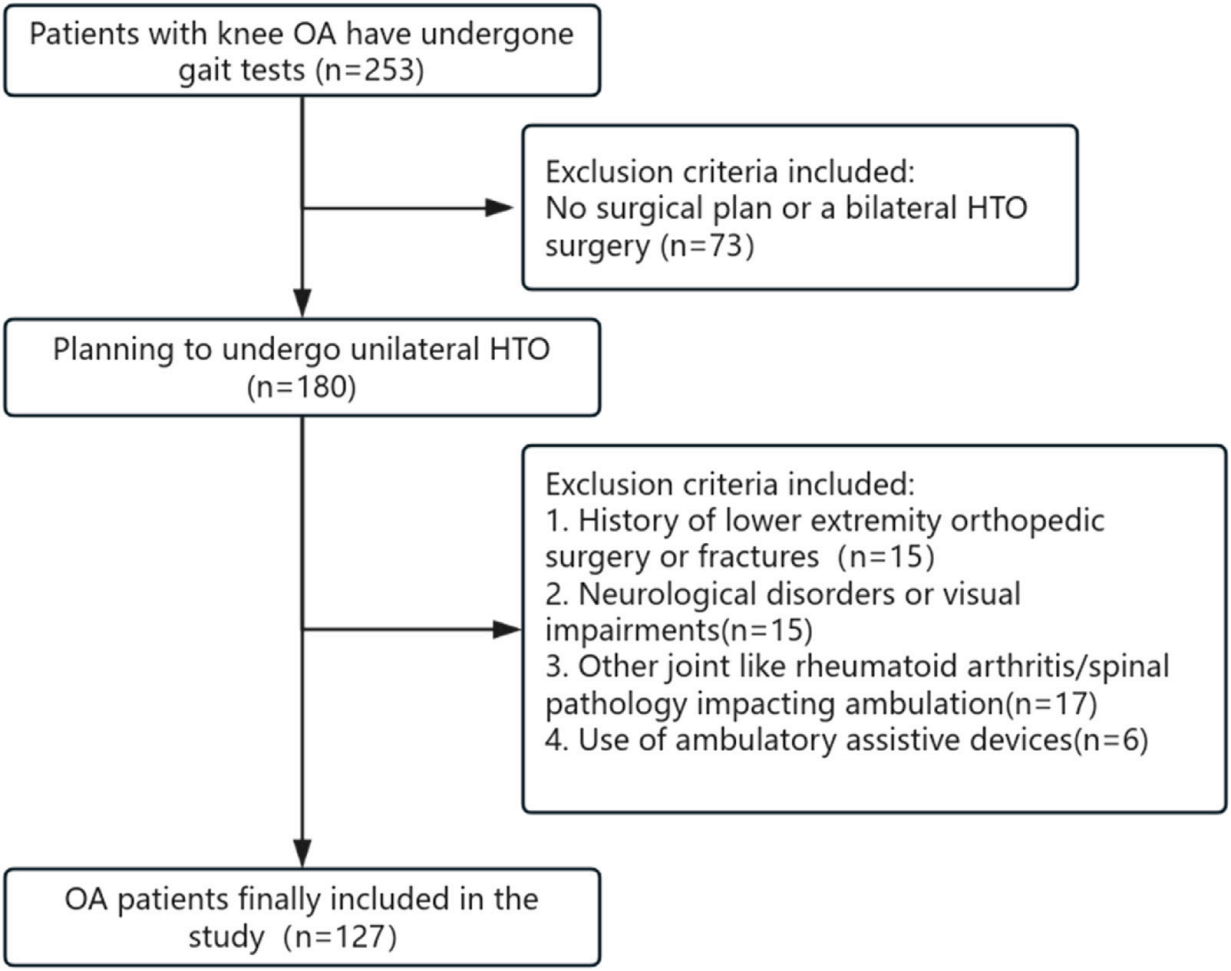
Flowchart of inclusion and exclusion criteria for patients with knee OA.

This retrospective study enrolled consecutive patients scheduled for unilateral high tibial osteotomy (HTO) between June 2019 and November 2021. Participants underwent gait analysis within 14 days before surgery. Inclusion criteria comprised: (1) diagnosis of knee OA meeting American College of Rheumatology classification criteria, (2) scheduled for unilateral HTO. The subjects included in this study were all those who underwent unilateral HTO surgery and who, after clinical assessment, had no surgical plans for the contralateral limb (Kellgren-Lawrence grade, K&L). The surgical knee was considered the more affected side, while the non-surgical limb was considered the less affected or unaffected contralateral side (Milner, 2008; Aljehani et al., 2019). Exclusion criteria included: (1) neurological disorders or visual impairments; (2) concomitant joint/spinal pathology impacting ambulation; (3) history of lower extremity orthopedic surgery or fractures; (4) use of ambulatory assistive devices (Figure 1).

The final cohort comprised 127 knee OA patients (n = 79 females, n = 48 males; female:male ratio 1.6:1). The study complied with the Declaration of Helsinki, receiving ethical approval from the Institutional Review Board of the Hospital (No. 2022-165). For this retrospective analysis, written informed consent was waived under institutional guidelines.

### Equipment

FPA was recorded using a Footscan^®^ pressure plate system (RSscan International, Olen, Belgium). The platform dimensions were 2093 × 469 × 18 mm, containing 16,384 resistive sensors configured in a 256 × 64 matrix with a sampling frequency of 126 Hz. The system was interfaced to a computer. During barefoot walking trials, ground reaction data were transmitted to proprietary Footscan^®^ software for processing. FPA and gait velocity were automatically computed by the software (Sharifi et al., 2024; Askari and Esmaeili, 2021).

### Procedure of testing toe-out gait

Before data collection, participants’ anthropometric data (including height and weight) were entered into the Footscan^®^ software. All participants received standardized instructions and performed practice trials walking along the platform to familiarize themselves with the protocol prior to formal testing.

During formal testing, each participant walked barefoot on the 2-m pressure plate at a self-selected comfortable speed without assistance. To minimize fatigue effects, 3-min rest periods were provided between each measurement. Three valid trials were selected per participant, and averaged for statistical analysis minimizing systematic errors. Gait parameters were automatically computed by the integrated software.

### Radiographic analysis

All participants underwent full-length weight-bearing anteroposterior (AP) radiography of both lower limbs within 14 days prior to gait analysis. Knee alignment and deformity severity were assessed using two parameters: hip-knee-ankle angle (HKA) and anatomical medial proximal tibial angle (aMPTA). The HKA angle is measured as the angle between the femoral mechanical axis (from the center of the femoral head to the center of the intercondylar fossa of the femur) and the tibial mechanical axis (from the center of the tibial intercondylar eminence to the center of the ankle joint); the aMPTA is the medial angle between the tangential line of the tibial plateau and the tibial anatomical axis (the central axis of the tibial diaphysis). Knee OA severity was graded in all subjects using the K&L classification system.

The radiographic measurements of HKA and aMPTA were performed by two independent orthopaedic surgeons. The final values for HKA and aMPTA used in statistical analyses were averaged from the measurements obtained by these two surgeons. K&L grading was performed and recorded by a single investigator (an orthopaedic surgeon with over 10 years of experience) for the knee OA subjects.

### Relevant risk factors

Potential risk factors associated with FPA patterns were selected based on clinical rationale and existing evidence. Six factors were analyzed: sex, age, body mass index (BMI), gait velocity, HKA, aMPTA, and K&L grade. These encompassed: (1) demographic factors (sex, age, BMI); (2) spatiotemporal gait parameters (gait velocity); (3) structural alignment measures (HKA, aMPTA) evaluating knee deformity extent; and (4) disease severity (K&L grade of affected side).

### Statistical analysis

Statistical analyses were performed using SPSS 25.0 (IBM Corp., Armonk, NY). Categorical data are presented as frequencies (percentages), continuous variables as mean ± standard deviation (SD).

The Shapiro-Wilk test assessed normality of the difference in FPA between affected and contralateral limbs and other continuous variables. Paired Student’s *t*-tests were used for normally distributed FPA comparisons between limbs, with Wilcoxon signed-rank tests for non-normally distributed data. In addition, we used the intraclass correlation coefficient (ICC) to assess the consistency of HKA and aMPTA measurements, with an ICC >0.75 indicating high consistency.

To evaluate the clinical relevance of FPA asymmetry in knee OA, adjusted odds ratios (ORs) for asymmetry risk factors were calculated using binary logistic regression. Participants were stratified by asymmetry pattern: AAL group: FPA of affected > FPA of contralateralCAL group: FPA of contralateral > FPA of affected.

Univariate screening employed independent samples t-tests or Mann-Whitney U tests for continuous variables, and chi-square tests for categorical variables. To retain potential predictors, variables with *P* < 0.1 were included in the multivariable logistic regression model. Using the CAL group as reference, ORs with 95% CIs estimated the likelihood of AAL group membership. Statistical significance was set at *P* < 0.05.

## Results

The final cohort comprised 127 knee OA patients (48 males, 79 females; male-to-female ratio 1:1.65). Mean age was 58.5 ± 7.9 years and mean BMI 27.3 ± 3.5 kg/m^2^. Affected limbs included 70 left and 57 right limbs (55.1% vs 44.9%). Detailed baseline characteristics of the cohort are presented in Table 1. The results of the consistency analysis showed that the ICC for the HKA angle among observers was 0.798, and the ICC for the aMPTA was 0.787, both of which were greater than 0.75.

**TABLE 1:** Participants’ characteristics (n = 127).

Variables	Mean ± SD/n (%)
Age,years	58.46 ± 7.94
Gender	
Male	48 (37.8%)
Female	79 (62.2%)
Height (m)	1.63 ± 0.08
Mass (kg)	72.95 ± 11.84
BMI (kg/m^2^)	27.31 ± 3.51
HKA (°)	171.70 ± 3.56
aMTPA (°)	84.68 ± 2.82
Affected K&L Grade	
Grade 1	2 (1.6%)
Grade 2	30 (23.6%)
Grade 3	58 (45.7%)
Grade 4	37 (29.1%)
Non-surgical K&L Grade	
Grade 0	27 (21.3%)
Grade 1	64 (50.3%)
Grade 2	30 (23.6%)
Grade 3	6 (4.7%)
Affected limbs	
Left	70 (55.1%)
Right	57 (44.9%)

HKA, hip-knee-ankle; aMPTA, anatomical medial proximal tibial angle; K&L, Kellgren-Lawrence grade.

**FIGURE 2:**
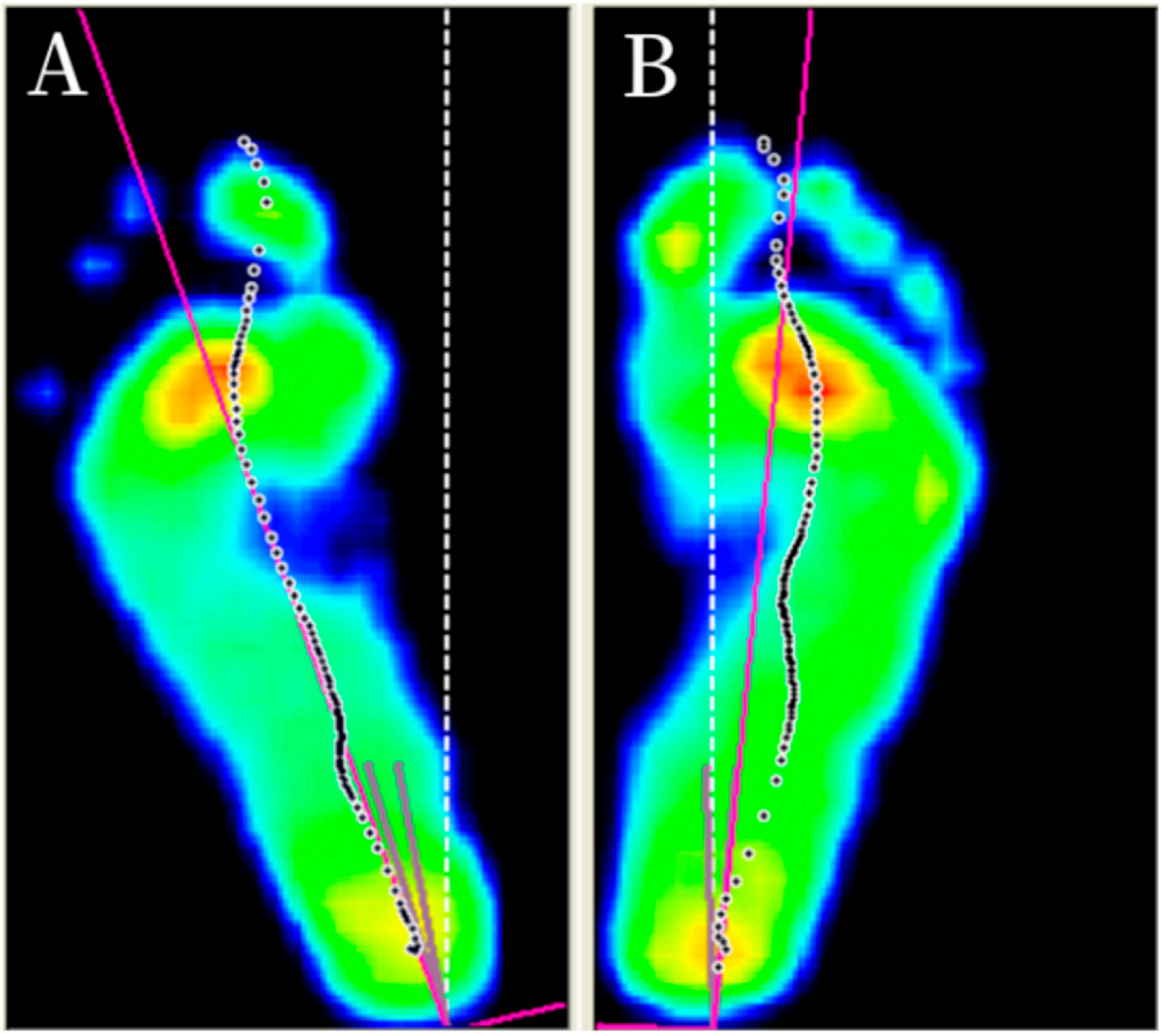
Measurement of foot progression angle (FPA) asymmetry FPA is defined as the angle between the foot longitudinal axis (red line) and the walking direction (white line). **(A)** Contralateral limb demonstrating larger FPA **(B)** Affected limb demonstrating smaller FPA.

Table 2 presents FPA comparisons between limbs in knee OA patients. FPA was significantly larger in contralateral limbs compared with affected limbs (15.21° ± 7.72° versus 11.38° ± 8.13°; *P* < 0.001; Figure 2). Table 3 details univariate analysis for FPA asymmetry patterns. K&L grade of affected side was significantly different across the AAL and CAL groups (*P* < 0.05). No between-group differences existed for age, sex, BMI, HKA, aMPTA, non-surgical K&L grade, or gait velocity (all *P* > 0.1).

**TABLE 2:** Comparision of FPA angle between two limbs in patients with knee OA (n = 127).

Group	FPA(°, Mean ± SD)	Mean difference (95%CI)	t value	*p* value
Affected limb	11.38 ± 8.13	−3.84 (5.33–−2.36)	−5.117	*p* < 0.001
Contralateral limb	15.22 ± 7.72			

FPA, foot progression angle.

**TABLE 3:** Univariable analysis of FPA asymmetry between AAL and CAL.

Variables	AAL (n = 39)	CAL (n = 88)	Z/t	P
Age, years	59.03 ± 7.65	58.20 ± 8.10	t = −0.191	0.848
Gender				
Male	16 (33.33%)	32 (66.7%)	χ^2^ = 0.250	0.693
Female	23 (29.1%)	56 (70.9)		
BMI, kg/m^2^	27.48 ± 3.15	27.23 ± 3.68	t = 0.366	0.715
aMPTA (°)	84.74 ± 2.28	84.66 ± 3.04	t = −0.084	0.933
HKA (°)	171.63 ± 3.49	171.73 ± 3.62	t = −0.350	0.726
Affected K&L GradeK&L grade				
K&L = 1/2	11 (28.2%)	21 (23.9%)	χ^2^ = 8.693	0.024*
K&L = 3	23 (59.0%)	35 (39.8%)		
K&L = 4	5 (12.8%)	32 (36.4%)		
Non-surgical K&L grade				
K&L = 0/1	28 (71.8%)	63 (71.6%)	χ2 = 0.653	0.721
K&L = 2	10 (25.6%)	20 (22.7%)		
K&L = 3	1 (2.6%)	5 (5.7%)		
Gait velocity (m/s)	0.79 ± 0.15	0.79 ± 0.19	t = 0.121	0.904

AAL, Larger FPA on affected limb; CAL, Larger FPA on contralateral limb.

*Significance: *p < 0.05.

Multivariable binary logistic regression analysis (adjusted for variables with P < 0.1 in univariate analysis. Table 4 show that Using K&L grade 4 as the reference category, Patients with K&L grade 1 or 2 OA had significantly lower odds of exhibiting the contralateral-larger FPA pattern (CAL group) compared to those with grade 4 OA (adjusted OR = 0.298, 95% CI: 0.091–0.982; P = 0.047). Similarly, patients with K&L grade 3 OA also had significantly lower odds of being in the CAL group compared to grade 4 patients (adjusted OR = 0.238, 95% CI: 0.081–0.700; P = 0.009). The overall model was statistically significant (Omnibus test: χ^2^ = 8.260, df = 2, P = 0.016) and demonstrated good fit (Hosmer-Lemeshow test: χ^2^ < 0.001, P = 1.000).

**TABLE 4:** Multivariable logistic regression analysis of factors associated with FPA asymmetry pattern (reference: CAL group**)**.

Variables	*B*	S.E.	Wald value	Or	Or (95%CI)	*p*
K&L grade			6.871			0.032
Grade 1/2	−1.210	0.608	3.957	0.298	(0.091,0.982)	0.047*
Grade 3	−1.436	0.551	6.803	0.238	(0.081,0.700)	0.009**
Grade 4(Ref)						
Constant	1.856	0.481	14.901	6.400		*p* < 0.001***

*CAL, Contralateral limb larger FPA (reference group). OR, Odds ratio for CAL group membership vs AAL group. AAL was assigned a value of 1 and CAL was assigned a value of 2. OR, odds ratio; S.E., standard error; Nagelkerke *R* ^2^ = 0.107; goodness of fit: Hosmer-Lemeshow X^2^ < 0.001, degree of freedom = 1, P = 1.000; model test: Omnibus test: X^2^ = 8.260, degree of freedom = 2, P = 0.016. Significance: **p < 0.05, ***p < 0.01, **p < 0.001.

## Discussion

The principal finding of this study is that patients with knee OA scheduled for unilateral HTO exhibit significant asymmetry in FPA, characterized by a larger FPA on the contralateral (less affected or unaffected) limb compared to the affected limb. Furthermore, this specific asymmetry pattern (contralateral limb larger FPA) was positively associated with higher radiographic disease severity (K&L grade) in the affected knee.

Increasing the FPA of the affected limb is often considered an effective gait correction method to reduce the KAM (Chang et al., 2007; Reeves and Bowling, 2011; Simic et al., 2013). The biomechanical principle behind this is adjusting the joint moment arm to decrease the adduction moment. Most previous studies only reported the FPA of the affected limb, with a lack of data on the FPA of the contralateral lower limb. However, our study describes and investigates the FPA gait patterns of both lower limbs in knee osteoarthritis patients without any artificial intervention. Our findings suggest that during daily walking, knee osteoarthritis patients may have a natural adaptive mechanism in the FPA gait of their two lower limbs, specifically showing a larger FPA on the contralateral limb. This might be related to the contralateral limb compensatively bearing more load.

The asymmetric FPA gait pattern identified in this study may stem not only from mechanical constraints caused by varus deformity, but also from active pain-avoidance behaviors. Previous studies have reported that due to pain and functional impairment caused by unilateral knee OA, the neuromuscular system undergoes adaptive changes, making the body tend to adopt load patterns that reduce the burden on the affected limb (Shakoor et al., 2003; Verlaan et al., 2018). The asymmetric FPA observed in our study (a larger foot progression angle on the contralateral side) may be part of such an adaptive gait pattern. It might aim to reduce the load on the affected limb, including the knee adduction moment, or improve walking stability. However, it must be emphasized that this compensatory adaptation also increases the load on the contralateral limb during daily walking. In addition, some scholars have reported that the reduced FPA on the affected side may be related to limited knee extension in OA (Hall et al., 2015).

Foot characteristics may also play an important role in the mechanical mechanisms of knee OA. Recent studies link pes planus, rearfoot eversion, reduced arch height to altered knee loading and OA progression (Maryam Sohrabi, 2024; Patil et al., 2024), with excessive pronation or collapsed medial arches shifting foot pressure centers, affecting lower limb alignment and joint load (Nakazato et al., 2025; Karatas and Utkan, 2024). This suggests foot-knee biomechanical interactions in advanced OA. Our observed bilateral FPA asymmetry may reflect OA-induced compensatory changes: greater contralateral FPA could counter medial loading, complementing neuromuscular compensation. Thus, FPA asymmetry is likely multifactorial, involving neuromuscular strategies and foot morphology, needing further study.

The significant association between higher K&L grade and greater likelihood of exhibiting the contralateral-larger FPA asymmetry suggests that this gait adaptation becomes more pronounced as the structural severity of knee OA increases. This aligns with studies linking higher K&L grades to increased knee adduction moment (Chen et al., 2021; Foroughi et al., 2009; Sharma et al., 1998; Wu et al., 2022), which is a key biomechanical surrogate for disease progression. The progressive nature of the observed gait asymmetry may reflect the body’s escalating compensatory demands in response to worsening joint pathology and pain.

Notably, walking speed, along with other factors like age, gender, BMI, HKA, and aMPTA, did not show a significant association with the specific FPA asymmetry pattern in our analysis. This suggests that while walking speed may be reduced overall in knee OA due to pain or other factors (Khan et al., 2017), it does not appear to be a primary driver of the asymmetrical FPA distribution between limbs observed here.

Additionally, our research noted that knee OA patients naturally assumed a toe-out gait of approximately 10° on both lower limbs without external intervention. Therefore, the results of this study do not suggest that all knee OA patients should blindly adopt gait modification to increase their FPA. As Uhlrich et al. emphasized, FPA-based gait intervention is highly dependent on the magnitude of baseline FPA. Adjusting gait according to the initial FPA angle is more effective in reducing the peak knee adduction moment than adjusting to a fixed angle (Uhlrich et al., 2018). These adjustments also hold promise in decelerating the advancement of knee OA in the medial compartment (Chang et al., 2007). Thus, gait modification must follow the principle of individualization: it is essential to assess a patient’s baseline FPA first before determining whether they are suitable for improving their condition by adjusting toe-out.

The present study has a few limitations. Firstly, the retrospective design limited the availability of some potentially relevant clinical data, such as patient-reported pain and function scores, which might offer deeper insights into the mechanisms and functional implications of FPA asymmetry. Secondly, our cohort consisted exclusively of patients with end-stage knee OA (K&L grade predominantly 3 or 4) scheduled for unilateral HTO. Consequently, the findings may not be generalizable to individuals with mild or moderate knee OA. To further validate similar biomechanical associations in early-stage OA, future studies focusing primarily on early-stage OA patients will be necessary.

## Conclusion

In conclusion, patients with advanced unilateral knee OA exhibit a characteristic asymmetric FPA during gait, manifesting as a larger FPA on the contralateral limb compared to the affected limb. This asymmetry pattern likely arises from a combination of compensatory neuromuscular adaptations and biomechanical constraints associated with the osteoarthritic knee (potential flexion contracture). Crucially, the presence and prominence of this specific asymmetry were significantly associated with higher radiographic disease severity (K&L grade) in the affected knee. These findings highlight the importance of considering limb-specific and disease severity-dependent gait adaptations in the biomechanical management of knee OA.

## Data Availability

The original contributions presented in the study are included in the article/supplementary material, further inquiries can be directed to the corresponding authors.
